# The Patterns of Altitudinal Gradient Differentiation in the Morphological Traits of *Calliptamus italicus* (L.) (Orthoptera: Acridoidea) and Their Environmental Driving Mechanisms in the Desert Steppe in the Ili River Basin

**DOI:** 10.3390/insects17050445

**Published:** 2026-04-22

**Authors:** Adilaimu Abulaiti, Huaxiang Liu, Xiaofang Ye, Hongxia Hu, Xuhui Tang, Yanxin Yang, Tiantian Wu, Shiya He, Fei Yu, Rong Ji, Roman Jashenko, Jie Wang, Huixia Liu

**Affiliations:** 1 Key Laboratory of Special Environment Biodiversity Application and Regulation in Xinjiang, International Center for the Collaborative Management of Cross-Border Pests in Central Asia, College of Life Sciences, Xinjiang Normal University, Urumqi 830017, China; 13579926930@163.com (A.A.); lhx184175@163.com (H.L.); 13579261461@163.com (X.Y.); huhongxia111@126.com (H.H.); 202214034058@stu.xjnu.edu.cn (X.T.); 202311014101@stu.xjnu.edu.cn (Y.Y.); 202311014104@stu.xjnu.edu.cn (T.W.); 202311014100@stu.xjnu.edu.cn (S.H.); yufei@xjnu.edu.cn (F.Y.); 2 Tacheng Research Field (Migratory Biology), Observation and Research Station of Xinjiang Tacheng, Tacheng 834700, China; 3 Changji University Office, Changji University, Changji 831100, China; jirong@xjnu.edu.cn; 4 Institute of Zoology, Ministry of Education and Science of Kazakhstan, Almaty 050038, Kazakhstan; roman.jashenko@zool.kz; 5 Urumqi Meteorological Bureau, Urumqi 830063, China; wj220466@163.com; 6 Urumqi Pastoral Meteorological Experimental Station Central Tianshan Grassland Ecology Monitoring Laboratory, Urumqi 830063, China

**Keywords:** morphological traits, altitudinal gradient, *Calliptamus italicus*, desert steppe, Ili River Basin

## Abstract

Morphological traits, as core components of functional traits, are fundamental in determining environmental adaptability. However, under climate warming, the adaptive morphological changes and associated ecological risks of locust populations migrating to higher altitudes remain poorly understood. Here, we investigated *Calliptamus italicus*, the dominant locust species in the desert steppes of the Ili River Basin, to explore the response patterns of its morphological functional traits along an altitudinal gradient and their relationships with environmental factors. Morphological measurements revealed that forewing area, width, and length, as well as hindwing width, exhibited highly significant positive correlations with altitude (*p* < 0.01); in contrast, body length, head width, head height, pronotum length, pronotum width, hind femur length, and hind tibia length displayed significant negative correlations with altitude (*p* < 0.05). All morphological indicators presented highly significant sexual dimorphism (*p* < 0.001). Ratio analysis showed that the pronotum width-to-head width ratio (M/C), pronotum height-to-head width ratio (H/C), and forewing length-to-hind tibia length ratio (E/F) were significantly positively correlated with the altitudinal gradient (*p* < 0.05), with all ratios exhibiting significant sexual differences (*p* < 0.05). Random Forest analysis showed that PC1 (75.5% of variation) reflected traits for feeding, jumping, and reproduction, whereas PC2 (5.6%) represented flight-related traits, with significant sexual dimorphism. This study demonstrates that trait variation in *C. italicus* along an altitudinal gradient is closely linked to environmental factors. Our findings provide critical data for predicting habitat adaptation responses in locust populations, thereby enhancing the precision and efficacy of locust plague management and contributing to the conservation and restoration of desert steppe ecosystems.

## Simple Summary

Morphological traits are fundamental in determining environmental adaptability, yet how climate warming drives adaptive morphological changes in locusts migrating to higher altitudes, and the associated ecological risks, remain poorly understood. We investigated *Calliptamus italicus* along an altitudinal gradient in the Ili River Basin to explore trait response patterns and environmental relationships. Forewing area, width, and length, as well as hindwing width, showed highly significant positive correlations with altitude (*p* < 0.01). Conversely, body length, head width, head height, pronotum length and width, hind femur length, and hind tibia length exhibited significant negative correlations (*p* < 0.05). All traits displayed significant sexual dimorphism (*p* < 0.001). Ratios of pronotum width to head width (PW/C), pronotum height to head width (PH/C), and forewing length to hind tibia length (E/T) increased significantly with altitude (*p* < 0.05). Principal component analysis revealed PC1 (75.5% variation) reflected feeding and jumping traits, while PC2 (5.6%) represented flight-related traits. This study demonstrates that trait variation along altitudinal gradients is closely linked to environmental factors, providing critical data for improving locust management and desert steppe conservation.

## 1. Introduction

Locust plagues rank among the world’s most significant natural disasters, posing a grave threat to grassland ecosystems and agricultural and pastoral production [1,2]. Locust population dynamics are closely linked to climate change and the stability of grassland ecosystems [3]. In recent years, alongside global warming, locust habitats have gradually expanded to higher altitudes. Coupled with the frequent occurrence of extreme droughts and floods, this has created favourable conditions for large-scale locust outbreaks [4,5]. Xinjiang (China), one of the regions with frequent locust plagues in China, has suffered locust-infested areas of 4 × 10^6^ hm^2^ [6], resulting in direct and indirect economic losses amounting to hundreds of millions of yuan, according to the Regional Classification of Dominant Locust Species in Xinjiang Grasslands. Among the dozen or so primary locust species causing damage, the *C. italicus*, the *C. barbarus*, and the *Locusta migratoria* are particularly prominent. The *C. italicus*, in particular, has repeatedly caused large-scale outbreaks in Northern Xinjiang and Central Asia due to its strong migratory and dispersal capabilities, making it a species of regional concern [3,7,8].

Functional traits constitute key phenotypic characteristics reflecting an organism’s adaptability to its environment, encompassing multiple dimensions including morphology, physiology, and behaviour [4,7]. Morphological traits, as the core constituents of functional traits [9], can effectively indicate species’ response strategies to environmental changes through intuitive phenotypes such as body size and organ proportions. They reveal adaptive strategies concerning resource utilisation, dispersal, and reproduction [7,10]. In orthopteran insects, body size directly correlates with metabolic demands, feeding adaptability, locomotive capacity, and dispersal potential, serving as a pivotal entry point for deciphering species’ environmental adaptation strategies [7,10]. Research indicates that under drought stress conditions, the body size of locusts exhibits significant synergistic changes with the composition of hydrocarbons in their cuticle. The concurrent reduction in body size and increase in cuticle hydrocarbon content under drought stress forms a dual defence strategy for water retention and drought resistance [11,12]. Moreover, female body length and abdominal width correlate positively with egg-laying capacity [13]. The robustness of the male hind femur length significantly influences mating success rates. This in-depth analysis of the relationship between morphology and function provides a crucial entry point for understanding the environmental adaptation mechanisms within locust populations [12].

The *C. italicus*, as a primary pest of Xinjiang’s grasslands, poses a severe threat to regional ecology and livestock farming due to its high migratory capacity and prevalence [3,14,15]. Global warming is further driving the expansion of its suitable habitat towards higher altitudes. As the most integrative environmental gradient within desert steppe ecosystems, altitude exerts directional selective pressures on the morphological evolution of ectothermic insects by linking gradient changes in factors such as temperature, humidity, and vegetation distribution [16]. Research has demonstrated that the *C. italicus* exhibits strong heat tolerance and is a typical low-altitude species. Wang et al. (2014) found that over the past 53 years, climate change has shifted the habitat characteristics of the *C. italicus*’s suitable range from warm and dry to warm and humid [17,18]. The findings of Wu et al. further confirm that the *C. italicus* exhibits its widest distribution within the altitude range of 1018.04–1961.65 masl, with its upper distribution limit currently rising at a rate of approximately 50 metres per decade [19]. This aligns closely with the trend of warming at high altitudes and a reduction in extreme cold events, suggesting that it may expand its ecological niche through phenotypic adaptation or genetic variation. Current research on the *C. italicus* primarily focuses on physiological and genetic aspects. Song et al. (2021) found that wintering eggs from high-altitude populations contained higher levels of cryoprotectants and exhibited a lower cold tolerance threshold [20]. Ren et al. (2015) demonstrated that high-altitude adults exhibit prolonged cold survival times and reduced upper limits of heat tolerance, with this physiological strategy laying the foundation for their dispersal to higher elevations [11]. Genetic studies have also revealed frequent gene exchange among populations of *C. italicus* at different elevations. Analysis based on the mitochondrial *Cytb* gene indicates weak genetic differentiation between populations, with total gene flow reaching as high as 13.84. This high level of gene flow effectively mitigates the genetic differentiation that geographical isolation might otherwise induce [21], primarily because the altitude gradient integrates the synergistic changes in multiple factors such as climate, vegetation, and soil [3,5,22]. As soil types shift and vegetation evolves from arid grassland communities to diverse assemblages, this multifactorial gradient exerts sustained selective pressure on the *C. italicus*. This compels it to adapt morphologically to its environment [23].

Present research on the morphological traits of the *C. italicus* exhibits significant limitations: firstly, relevant findings remain scarce, with particularly insufficient targeted studies on the patterns of morphological differentiation and environmental response mechanisms. Against the backdrop of global climate change, the fluctuating characteristics of extreme temperatures are especially pronounced. For example, the annual mean temperature in China’s Pearl River Delta region increased by up to 0.49 °C per decade between 1960 and 2011, nearly double the global average for land surfaces (0.21 °C per decade) [24,25]. Moreover, existing research has confirmed that extreme temperatures exhibit heightened sensitivity to climate variability, and there exists a direct correlation between species’ morphological trait variation and extreme temperature events [26]. This pattern is more pronounced in the arid and semi-arid regions of Xinjiang, while the Yili River Basin, as a typical climate-sensitive region, has shown a particularly prominent warming trend in recent years [22,27]. The drastic climate changes render the conclusions drawn in 2011 inadequate to reflect the current situation. Second, existing studies have not covered the continuous altitude gradient of 700–1700 masl, and the dynamic variation patterns of core functional traits such as wing morphology and body size remain to be systematically analysed.

As the core pasture for rotational grazing in Xinjiang’s arid and semi-arid steppe during spring and autumn, the desert steppe has long been under high grazing pressure, leading to prominent grassland degradation [8]. However, it is also the dominant habitat with the highest population density of the *C. italicus*. Its drought-tolerant and barren-resistant characteristics not only meet the physiological needs of *C. italicus* for low temperatures, drought, and sparse vegetation, but also provide an ideal habitat for the locust to feed and reproduce more easily due to the simplified vegetation structure caused by grassland degradation. Ultimately, this forms a highly adaptive relationship between degraded grasslands and the outbreak of *C. italicus* [28]. Consequently, our research focuses on the *C. italicus*, the dominant species within desert steppes in the Ili River Basin. We aimed to systematically investigate morphological and functional trait variations along a continuous altitudinal gradient, with three core objectives: (1) to clarify the response patterns of morphological traits and their ratios in *C. italicus* along the altitudinal gradient; (2) to elucidate the sex-specific nature of morphological differentiation between males and females and its adaptive significance at high altitudes; (3) identify core environmental factors driving morphological variation and elucidate the adaptive relationship between environment, morphology, and function. These findings will provide data support for understanding locust adaptation during high-altitude expansion and offer scientific evidence for sustainable management of desert grassland ecosystems.

## 2. Materials and Methods

### 2.1. Overview of the Study Area

**Figure 1:**
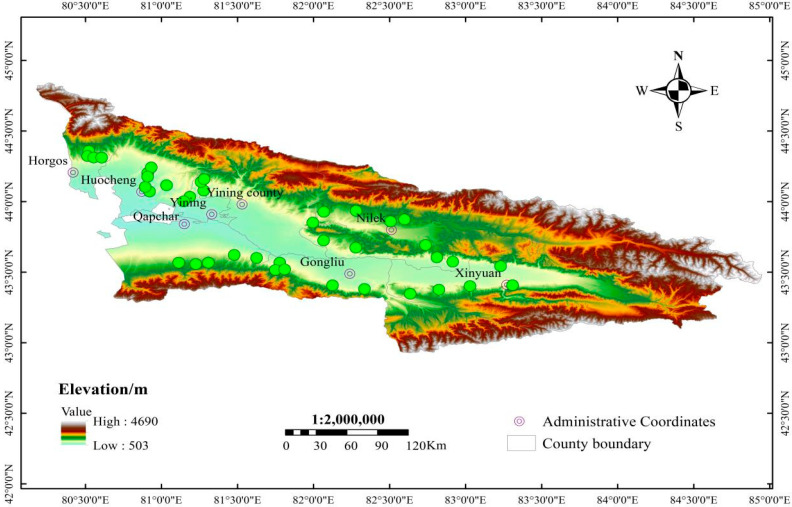
Location of study area. Green dots indicate sampling locations.

This study focused on a field survey of the *C. italicus* in the typical locust-infested desert grassland areas of the Ili River Basin (42°14′–44°50′ N, 80°09′–84°56′ E) (Figure 1). The total area is approximately 57,600 square km^2^. The Ili River Basin exhibits complex topography, primarily comprising basins, hills and plains. Not only does it feature a wide range of elevations and significant overall elevation differences, but it also contains numerous arid and ecologically fragile areas [29,30]. The region has an average elevation of 1064 masl, an annual mean temperature of 9 °C, and annual precipitation ranging from 200 to 800 mm, exhibiting a temperate continental arid–semi-arid climate [14,22].

This type of grassland accounts for 25–30% of the total grassland area within the watershed. The vegetation is dominated by xerophytic and hyper-xerophytic plants, primarily including *Seriphidium transiliense*, *Ceratocarpus arenarius*, *Sophora alopecuroides*, *Stipa capillata*, *Festuca ovina*, and numerous other species [2].

### 2.2. Research Methods

This study focused on the dominant species of locust in typical locust-prone areas of the Ili River Basin, the *C. italicus*, establishing a total of 40 sampling points across an altitude range of 700–1700 masl. At each site, a standard 100 m × 100 m plot was established. Five parallel sampling belts (2 m wide) were set diagonally within the plot, spaced 10 m apart to form a spatial gradient sequence. A 5 m buffer was retained between the belts and the plot boundary to reduce edge effects. Between May and September 2024, three sampling campaigns were conducted (including locust surveys, vegetation community investigations, and soil sample collection). Due to the locust occurrence period, May coincided with the hatching stage for *C. italicus*, with the vast majority being 1st to 3rd instar nymphs. The instability of morphological characteristics made species identification difficult, resulting in only nymph counts and adult *C. italicus* individuals being recorded. Consequently, May data were excluded. July marked the peak outbreak period for locusts. September represented the residual locust period, with both July and September exhibiting distinct locust morphological characteristics. Consequently, this study exclusively utilised survey data from July and September for subsequent analysis.

#### 2.2.1. Survey and Identification of Locust Species

This study employed standard sweep-net sampling to investigate locust communities to conduct locust community investigations [8]. Five equidistant transects were set at 50 m intervals at each sampling site. The sweep net was swept from left to right through a 180° arc along each transect at a constant speed of 0.5 m s^−1^ (one sweep constituted one net pass). Each set comprised ten net passes, with five sets collected per transect. The locust species and sex ratio were recorded within each set, and population density was converted to “individuals per ten net passes” [8,17,31,32]. Surveys were conducted on clear days with no rainfall and light winds, during the peak locust activity period between 10:00 and 18:00. Field records included collection sites and latitude and longitude coordinates, as well as habitat photographs [6,33]. Known locust species were kept alive in centrifuge tubes, whereas unknown species were placed in vials and transported to the laboratory for identification using the Fauna Sinica as a reference [34]. Adult locusts were captured randomly at each sampling site, with no fewer than 10 individuals of each sex (except in isolated plots). Each specimen was individually placed in a 10 mL cryogenic vial containing anhydrous ethanol for immediate preservation and transported to the laboratory for subsequent use [7].

Morphological traits were measured using an electronic vernier calliper with an international standard precision of 0.01 mm. Fifteen morphological characters indices were measured for 601 specimens: forewing area/width/length, hindwing area/width/length, body length, head width/height, pronotum length/width/height, hind tibia length/width, and hind tibia length. Each measurement was taken three times, with the mean value recorded. Wing area measurements were calculated via digital analysis using Photoshop software 2025 [7,8].

#### 2.2.2. Vegetation Survey and Assessment

Each 100 m × 100 m standard plot was surveyed using the five-point sampling method [8,35,36,37]. Five 1 m × 1 m herbaceous subplots were placed at the centre and one near each of the four corners (covering both core and fringe habitats). Within each plot, plant species names were recorded, the average natural height of the dominant layer was measured using a tape measure, and vegetation cover was recorded through visual estimation combined with grid method calibration [38]. Individual counts were taken to determine population density [37,39]. Following measurement of indicators, aboveground vegetation within the sample plots was cut flush with the ground surface. Total fresh weight was determined using an electronic balance accurate to 0.01 g. Samples were sorted by species and packed into breathable paper bags labelled with the plot number, species name, and sampling date. Then these samples were dried in an electric constant-temperature oven at 80° C for 24 h until constant weight was achieved, for subsequent dry biomass calculations [40]. All plant species within the sample plots were identified and taxonomically confirmed through specimen examination based on Flora of China [41]. Additionally, the Shannon–Wiener index (*H*′) and Pielou’s evenness index (*J*) were calculated for each sampling site. The specific formulas are as follows [2,8,37,39]:

The formula for calculating the Shannon–Wiener (*H*′) index is

(1) $$H^{\prime }=-\sum_{i=1}^{s}P_{i}\mathrm{Ln}P_{i}\;\;\;P_{i}\;=\;N_{i}/N$$

The formula for calculating the Pielou uniformity (*J*) index is

(2) $$J=H^{\prime }/\mathrm{Ln}S$$

In the formula, *S* denotes the number of species within each plot, *N* represents the total number of individuals across all species within the plot, and *P_i_* indicates the density of the *i*-th species.

#### 2.2.3. Soil Sampling and Analysis

Following completion of the ground vegetation survey within each plot, soil samples from the 0–10 cm layer were collected using a soil auger from the centre of each plot and thoroughly mixed [42]. After removing impurities such as stones and plant debris, the soil was air-dried naturally, then ground and sieved. Soil samples retained on the 1 mm sieve were used to determine pH, while those retained on the 0.25 mm sieve were used to measure soil electrical conductivity, salinity, total soluble solid content, organic matter, total nitrogen, and total phosphorus content [39,43,44]. Using a 100 cm^3^ standard ring mould, undisturbed soil samples were collected from this soil layer. These were dried at 105 °C to constant weight for calculating soil moisture content and bulk density [39,44].

#### 2.2.4. Acquisition of Environmental Factors

This study utilised ArcGIS 10.8 software to process historical climate data from 1957 to 2022 from 35 national benchmark meteorological stations in northern Xinjiang. The data, released by the National Meteorological Science Data Centre (http://www.cnern.org.cn), was processed based on the latitude and longitude coordinates of each field plot [45]; spatialisation of climate data was achieved through a Geographically Weighted Regression (GWR) model, combined with an Inverse Distance Weighted (IDW) interpolation algorithm, to generate continuous spatial raster data for annual precipitation, mean annual temperature, and mean annual wind speed within the study area. Terrain factors were extracted from 30-metre resolution Digital Elevation Model (DEM) data. Following geometric precision correction and coordinate system registration of the raw DEM data, the slope and aspect information for each sampling point was batch-extracted using the surface analysis tools within the 3D Analyst module of ArcGIS 10.8 software. These data were subsequently employed for statistical analysis [8,46,47].

### 2.3. Data Processing

This study employed Microsoft Excel for the preliminary processing of all data. Calculations of Pielou’s evenness index and the Shannon-Wiener index were performed using the “diversity” and “plyr” functions within the “vegan” package of R software version 5.1.2 [7,40]. Using SPSS 26.0 software, univariate analysis of variance was conducted on 15 functional traits and 5 morphological ratios in male and female individuals across different altitudinal gradients to test for significant differences in each trait across the altitudinal gradient (*p* < 0.05) [43]. Using Origin 2024 software, Linear Regression Analysis was employed to examine quantitative trends in morphological traits and their ratios along an altitudinal gradient. Principal component analysis (PCA) was then applied to extract the primary dimensions of variation (principal components) in morphological traits across the altitudinal gradient, revealing the overall pattern of trait synergistic responses to altitude. A Random Forest (RF) model was constructed using the randomForest package to analyse the explanatory power and importance of environmental factors on morphological principal components PC1 and PC2. The rfPermute package performed permutation tests to validate the statistical significance of model importance results. Data cleaning, filtering, and organisation were completed using the dplyr package within the tidyverse package, systematically revealing the response patterns of morphological principal components to environmental heterogeneity under the altitudinal gradient [2,7].

## 3. Results

### 3.1. The Response of Morphological Traits in the C. italicus Along an Altitudinal Gradient

**Figure 2:**
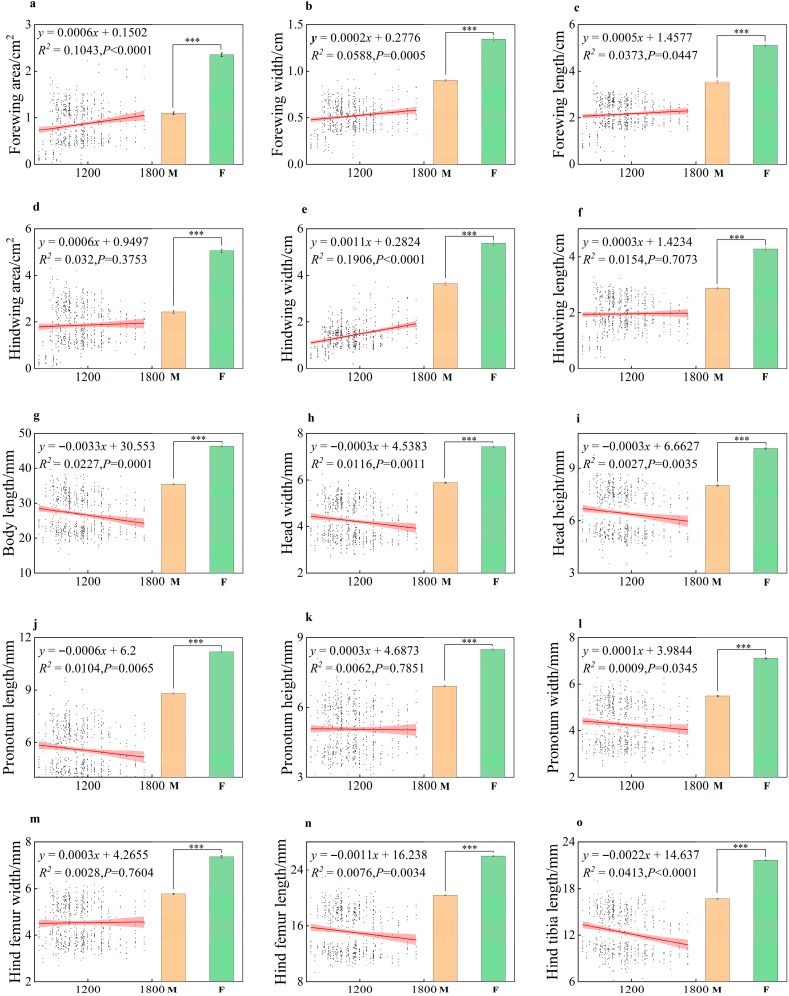
Linear relationships between 15 morphological traits of *C. italicus* along an altitudinal gradient (700–1700 m), and differences associated with sexual dimorphism (F: Female; M: Male). Green indicates females and orange indicates males. Red lines represent the linear fitting of each trait against elevation; *** *p* < 0.001 indicates a significant linear correlation. Bar plots inset in each subfigure show the female-to-male mean ratio for each trait, illustrating sexual dimorphism differences. (**a**): Forewing area; (**b**): forewing width; (**c**): forewing length; (**d**): hindwing area; (**e**): hindwing width; (**f**): hindwing length; (**g**): body length; (**h**): head width; (**i**): head height; (**j**): pronotum length; (**k**): pronotum width; (**l**): pronotum height; (**m**): hind femur width; (**n**): hind femur length; (**o**): hind tibia length.

Analysis of morphological traits revealed significant differentiation along the altitudinal gradient. With increasing altitude, forewing area, forewing width, forewing length, and hindwing width exhibited a marked increasing trend (*p* < 0.05), whereas body length, head width, head height, pronotum length, and hind tibia length showed a significant decreasing trend (*p* < 0.05). No significant differences were observed for other traits across the elevation gradient. At each elevation gradient, highly significant differences were found between male and female Italian locust individuals for all morphological traits (*p* < 0.001) (Figure 2). In summary, *C. italicus* morphological traits exhibited functionally dimension-specific responses along the elevation gradient: body size-related traits (body length, head width, etc.) significantly decreased, while wing-related traits (forewing area, forewing length, etc.) markedly increased. The highly significant variation in morphological ratios—pronotum height/head width (PH/C) and forewing length/hind femur length (FL/HF) (*p* < 0.001)—further underscores the pivotal role of enhanced flight function and optimised body proportions in high-altitude adaptation.

### 3.2. The Response of Morphological Trait Ratios in C. italicus Along an Altitudinal Gradient

**Figure 3:**
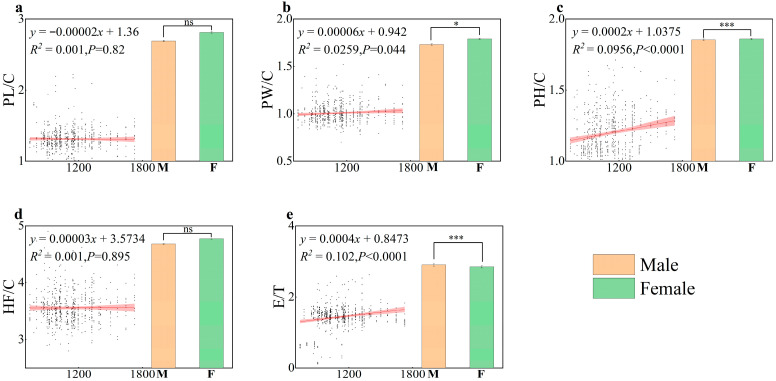
Sexual differences in 5 morphological trait ratios of *C. italicus*. (**a**) PL/C: pronotum length/head width; (**b**) PW/C: pronotum width/head width; (**c**) PH/C: pronotum height/head width; (**d**) HF/C: hind femur length/head width; (**e**) E/T: forewing length/hind femur length. “M” and “F” represent male and female individuals, respectively. Red fitted lines denote the distribution trend for each trait ratio. Significance levels are indicated as: ns (*p* > 0.05, not significant), * (*p* < 0.05, significant), *** (*p* < 0.001, highly significant). Bar plots in each subfigure show the mean values of each trait ratio for males and females.

Analysis of five morphological trait ratios (PL/C, PW/C, PH/C, HFL/C, and FL/HF) in female and male *C. italicus* revealed that the H/C and E/F ratios exhibited extremely significant variation (*p* < 0.001) along the altitudinal gradient, while the PW/C ratio showed significant variation (*p* < 0.05). Concurrently, significant sex-specific differences were observed for the PW/C, PH/C, and FL/HF morphological ratios between male and female individuals (Figure 3).

### 3.3. Principal Component Analysis of Morphological Traits in C. italicus Across Elevation Zones in Desert Grasslands

**Figure 4:**
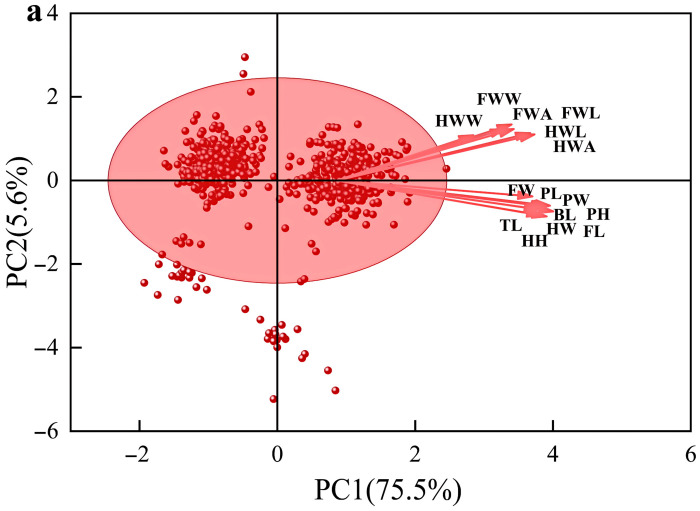

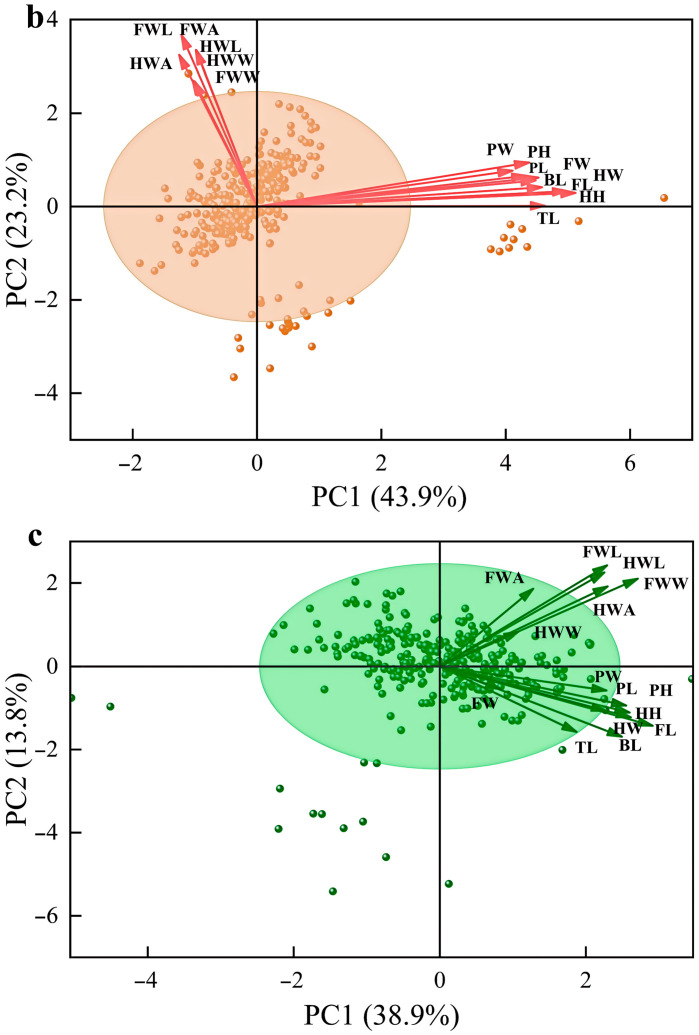
Principal component analysis of morphological traits in *C. italicus* across an elevation gradient in desert grasslands. (**a**–**c**) denote the morphological characteristics of the total, male, and female locust populations, respectively. FWA: forewing area; FWW: forewing width; FWL: forewing length; HWA: hindwing area; HWW: hindwing width; HWL: hindwing length; BL: body length; HW: head width; HH: head height; PL: pronotum length; PW: pronotum width; PH: pronotum height; FW: hind femur width; FL: hind femur length; TL: hind tibia length.

To further investigate the relationship and interactions between functional traits of male and female desert grassland locusts and altitude, this study conducted a principal component analysis on 15 morphological traits. Principal component 1 (PC1) was categorised as traits related to feeding, jumping, and reproduction (body length, head width, head height, pronotum length, pronotum width, pronotum height, hind femur length, hind femur width, and hind tibia length). Principal component 2 (PC2) encompassed traits related to flight (forewing area, forewing width, forewing length, hindwing area, hindwing width, and hindwing length) (Figure 4).

Analysis of the total locust population revealed (Figure 4a) that PC1 and PC2 contributed cumulative variance of 75.5% and 5.6% respectively, together explaining 81.1% of total morphological variation. Analysis of trait loadings on each principal component indicated that PC1 showed significant positive correlations with all measured morphological traits. In contrast, PC2 was primarily associated with forewing area, width, and length, as well as hindwing area, width, and length, predominantly indicating a functional dimension related to flight.

In the male locust population (Figure 4b), principal component analysis revealed that PC1 and PC2 contributed cumulative variance of 43.9% and 23.2% respectively, together explaining 67.1% of total morphological variation. Trait analysis indicated that PC1, in conjunction with (body length, head width, head height, pronotum length, pronotum width, pronotum height, femur length, femur width, and tibia length), explained dimensions related to feeding, jumping, flight capability, and reproduction. In contrast, PC2 was closely correlated with all morphological traits, indicating a functional dimension independent of overall body size, associated with body proportions and the development of localised structures.

Analysis of female *C. italicus* (Figure 4c) revealed that principal component axis 1 (PC1) and principal component axis 2 (PC2) contributed 38.9% and 13.8% of the cumulative variance, respectively, together explaining 52.7% of the total morphological variation. Trait analysis indicated that the correlation between PC1 and PC2 was consistent with that observed in the entire population.

### 3.4. The Response of Morphological Traits in the C. italicus to Environmental Factors

**Figure 5:**
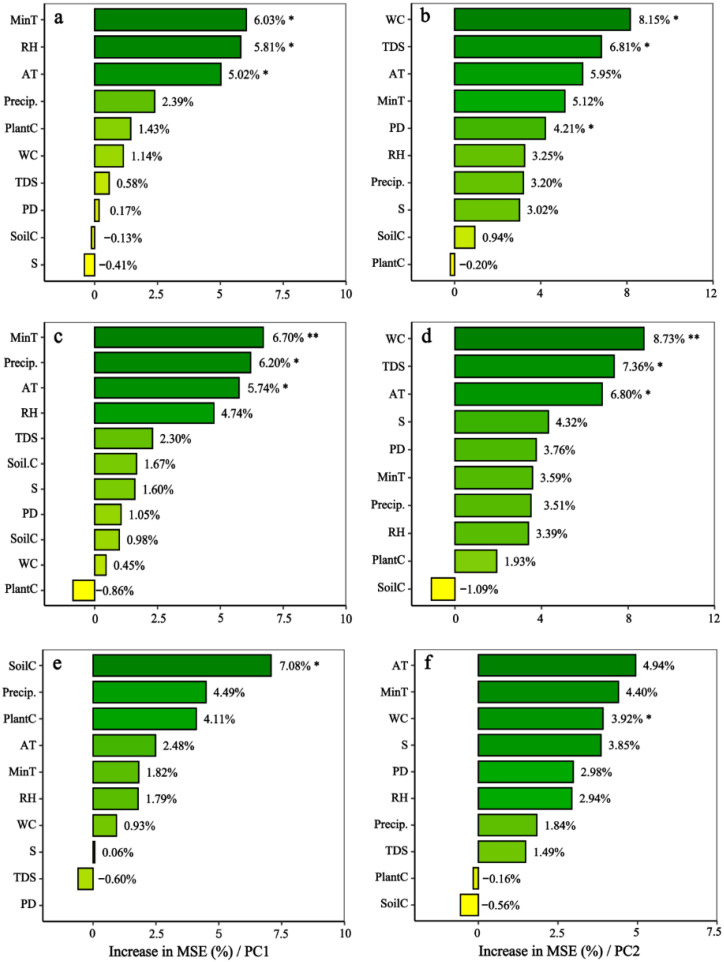
Key factors influencing the morphological traits of the *C. italicus*. (**a**,**b**) represent total *C. italicus* PC1/PC2, (**c**,**d**) represent male *C. italicus* PC1/PC2, and (**e**,**f**) represent female *C. italicus* PC1/PC2. AT: average temperature, MinT: minimum temperature, RH: relative humidity, Precip.: precipitation, S: salinity, TDS: total dissolved solids, WC: soil water content, PD: plant diversity; SoilC: soil carbon content, PlantC: plant carbon content. The color intensity of the green bars represents the magnitude of variable importance, with darker colors indicating greater importance. * indicates *p* < 0.05, and ** indicates *p* < 0.01.

The Random Forest (RF) model prediction results indicated that principal component 1 (PC1), explaining locust traits related to feeding, jumping, and reproduction, exhibited significant correlations with minimum temperature, average temperature, relative humidity, precipitation, and soil carbon content. Specifically, the total population’s PC1 showed significant correlations with average temperature and relative humidity (*p* < 0.05) (Figure 5a). The male population exhibited a highly significant correlation with minimum temperature (*p* < 0.001) and significant differences with minimum temperature, average temperature, and precipitation (*p* < 0.05) (Figure 5c); the female population showed significant differences with soil C (*p* < 0.05) (Figure 5e). PC2, explaining locust flight-related traits, showed significant correlations with soil moisture content, mean temperature, total soluble solids, and plant diversity. The total population exhibited significant differences in total soluble solids and plant diversity (*p* < 0.05) (Figure 5b); the male population showed a highly significant correlation with soil moisture content (*p* < 0.001) and significant correlations with average temperature and total soluble solids (*p* < 0.05) (Figure 5d); the female population exhibited a significant correlation only with soil moisture content (*p* < 0.05) (Figure 5f).

## 4. Discussion

### 4.1. The Response Patterns of Morphological Traits in the C. italicus Along Altitudinal Gradients and Their Adaptive Significance

This study focused on the desert steppe of the Ili River Basin, systematically analysing the differentiation patterns of 15 morphological traits in the *C. italicus* across an altitude gradient of 700–1700 metres. Findings reveal dual characteristics in its morphological responses: functional dimension specificity and sexual dimorphism. This provides crucial empirical evidence for understanding the morphological adaptation mechanisms underlying this species’ expansion into high-altitude regions.

Through further analysis of the association patterns between morphological traits and elevation gradients, the core morphological adaptive characteristics of the *C. italicus* at high elevations were clarified: body-related traits (e.g., body length) exhibited a significant decrease with increasing altitude (*p* < 0.05), whereas wing-related traits (e.g., forewing area) showed a significant decrease (*p* < 0.05). The morphological ratio of pronotum height/head width (PH/C) and forewing length/hind femur length (E/T) exhibited highly significant variation (*p* < 0.001). This result further quantifies an adaptive strategy optimising body proportions to enhance flight function. The differential response of these functionally related traits to elevation likely represents a key mechanism enabling this species to cope with high-altitude conditions, including low temperatures and spatially fragmented resources. As environmental conditions shift from high temperature and low humidity to low temperature and high humidity, locust wing types tend to increase in size, while average body size decreases with increasing altitude. This aligns with the conclusions proposed by Makarieva et al. (2005) in their review of Orthoptera insects, representing a synergistic adaptation to high-elevation cold temperatures and resource-heterogeneous habitats [48,49]. Specifically, a smaller body size reduces the surface area-to-volume ratio, thereby minimising heat loss under low-temperature conditions while shortening the developmental cycle to adapt to the brief growing season in high-altitude regions. Concurrently, reinforced wing structures optimise flight capabilities and enhance locomotive efficiency, enabling individuals to traverse habitats characterised by sparse vegetation and patchy resource distribution in high-altitude areas [33,50]. This morphological adaptation strategy converges with the cold adaptation mechanisms observed in Lepidoptera by Krishna et al. (2021): darker butterfly wings enhance solar radiation absorption efficiency to elevate body temperature [51]. As temperatures decrease, body colouration darkens; these dark wings enable butterflies to absorb more heat in colder climates. Similarly, the thoracic hairs or body bristles of high-altitude species conserve warmth by reducing heat loss [52]. All demonstrate morphological adaptations specifically geared towards low-temperature habitats.

Moreover, all 15 morphological traits examined in the *C. italicus* in this study exhibited highly significant sexual dimorphism (*p* < 0.001), consistent with findings from most studies on sexual size dimorphism (SSD) in orthopteran insects [53]. Specifically, females in low-altitude regions exhibited more developed reproductive traits such as body length and head width, enabling them to better meet the energy demands of ovarian development and reproductive activities. This phenomenon suggests that soil carbon content can enhance vegetation nutrient levels, thereby improving food quality and providing material support for the normal development of female reproductive traits [44,54]. The aforementioned findings indicate that male and female *C. italicus* develop distinct gender-specific adaptive pathways, confirming that sex serves as a key intrinsic factor regulating the expression of morphological traits and driving population differentiation within this species.

Elevation variation in morphological ratios further illuminates the species’ fine-scale adaptive mechanisms, revealing marked differences in both the magnitude and significance of variation among the different ratios: pronotum height/head width (PH/C) and forewing length/hind femur length (E/T) exhibited extremely significant increases with elevation (*p* < 0.001), whereas pronotum width/head width (PW/C) showed a significant increase (*p* < 0.05). The progressive increase in the PH/C and PW/C ratios indicates a relative reduction in head size and a corresponding broadening of the pronotum in high-elevation individuals. This adaptation may stem from the pronotum’s enhanced capacity to accommodate larger air sac structures, suggesting this feature facilitates improved oxygen storage and exchange efficiency within hypoxic environments [55]. The highly significant variation in E/T quantifies the functional trade-off between flight and jumping. Low-elevation individuals exhibit longer hind femur lengths, relying on potent jumping ability for movement within their habitat, adapted to the open and patchy landscapes of desert grasslands. High-elevation individuals display a greater proportion of forewings, utilising strong flight capabilities to traverse greater distances between resource patches. Previous studies indicate that within the 1200–2400 m elevation range, individual size within locust communities exhibits a pronounced U-shape distribution along the elevation gradient, with a size trough occurring around the 1700 m mesic zone [7]. This research conclusion is highly consistent with the observations of this study: the body size of *C. italicus* gradually decreases with increasing elevation, similarly reaching its minimum at approximately 1700 m. The differential response patterns of these morphological traits and ratios along the elevation gradient fundamentally represent the species’ long-term adaptation to heterogeneous habitat factors.

### 4.2. Key Environmental Drivers of Morphological Trait Differentiation

This study combined principal component analysis (PCA) and Random Forest (RF) models to systematically clarify the environmental driving mechanisms underlying the morphological trait differentiation of *C. italicus*. The core findings are as follows: morphological traits corresponding to different functional dimensions are dominated by distinct environmental factors, and this driving effect exhibits significant sexual specificity—a characteristic that fully reflects the species’ precise adaptation strategies to heterogeneous habitats.

Principal component analysis (PCA) revealed that PC1 corresponds to the feeding–jumping–reproduction functional dimension composed of head, pronotum, and hind leg traits, with its environmental driving characteristics exhibiting dual specificity at the group and sex levels. For the total locust community, low-temperature and humidity-related climatic factors are the core drivers of PC1—a regulatory logic explainable by habitat-adaptive mechanisms: low-temperature stress in high-elevation areas inhibits muscle development and reduces jumping ability [56], while the miniaturisation of head and hind leg traits decreases energy requirements, adapting to metabolic limitations under low-temperature conditions; simultaneously, the higher relative humidity in high-altitude areas alleviates drought stress, further reinforcing the adaptive trend of body size miniaturisation, which reflects the universal regulatory role of climatic factors on morphological traits. Sexual differentiation is closely associated with the niche differences between male and female individuals: the driving factors of PC1 in males include low temperature, precipitation, and average temperature, which are related to their wider activity range and the need to cope with more complex environmental fluctuations; in females, PC1 is mainly driven by soil carbon content. The core reason is that soil carbon content influences the availability of key nutrients, such as nitrogen and phosphorus, in vegetation, thereby providing energy support for reproduction-related traits such as ovarian development in females [44], highlighting the dominant influence of reproductive needs on the differentiation of female morphological traits.

PC2 corresponds to the flight functional dimension composed of forewings and hindwings, with its environmental driving dominated by soil and vegetation factors, and a clear top-down driving chain of “soil structure → vegetation distribution → flight traits” is formed. In the total community, soil water content, total soluble solids, and plant diversity are the key driving factors of PC2. Due to the habitat characteristics of the desert steppe in the Ili River Basin—such as high soil bulk density, water scarcity, and disturbance from overgrazing [57]—vegetation dwarfing and reduced food quality have occurred. To cope with this habitat constraint, locusts have evolved a phenotype of increased wing area to expand foraging ranges. Meanwhile, low plant diversity further promotes the improvement of flight ability, helping them cross resource patches. This adaptive strategy is consistent with the top-down effect theory proposed in relevant studies [58]. Sexual specificity is also significant in the flight functional dimension: PC2 in males is jointly driven by multiple soil and climatic factors, which matches the reproductive strategy of males needing to expand their mating range through flight. In contrast, PC2 in females is only driven by soil water content, which is highly compatible with their ecological characteristics of relatively fixed oviposition habitat selection and limited activity range.

The Random Forest (RF) model further validated and complemented the previously proposed driving mechanisms, confirming that the differentiation of morphological traits corresponding to PC1 and PC2 is not the result of the independent action of a single factor, but rather arises from the synergistic effects of multiple climate, soil and vegetation variables. In particular, the model supports a hierarchical pathway linking soil properties, vegetation distribution, and flight-related traits, thereby strengthening the ecological interpretation of the relationships between morphological traits of *C. italicus* and environmental factors.

## 5. Conclusions

The findings of this study reveal that the locust exhibits a characteristic pattern of “body size reduction coupled with enhanced locomotor organs” with increasing altitude. Body size-related traits show a significant decrease with altitude, while wing-related traits increase markedly. Morphological ratios reveal that high-altitude individuals adapt to hypoxia through broadened pronotums and enhanced flight capability via increased forewing proportion, while low-altitude individuals develop elongated hind legs for improved jumping. A size trough occurs at approximately 1700 m, consistent with locust community patterns. All morphological traits exhibit highly significant sexual dimorphism. Females show strong association with soil carbon to support reproductive demands, while males exhibit heightened sensitivity to low temperatures to accommodate broader foraging ranges. Sex emerges as the core regulator of morphological expression. Principal component analysis identified two functional dimensions: feeding–jumping–reproduction (PC1) and flight (PC2). Random Forest modelling confirmed PC1 is dominated by temperature and humidity, while PC2 is regulated by soil moisture content and plant diversity, forming a chain-driven mechanism linking soil–vegetation–insects. This indicates morphological differentiation results from the synergistic effects of climate, soil, and vegetation factors. This study elucidates the high-altitude adaptation mechanisms of the *C. italicus*, providing scientific reference for research on widespread locust species and locust plague control in Xinjiang grasslands. Subsequent investigations may employ molecular approaches to explore its genetic regulatory mechanisms and climatic adaptation potential.

## Data Availability

The original contributions presented in this study are included in the article/Supplementary Material. Further inquiries can be directed to the corresponding author.

## Supplementary Materials

The following supporting information can be downloaded at: https://www.mdpi.com/article/10.3390/insects17050445/s1, Table S1: Measured morphological characters of *Calliptamus italicus*. Table S2: Morphological-adaptive significance of the 15 measured traits in relation to environment. Table S3: Species composition and abundance of grasshoppers along the elevation gradient. Table S4: Species composition of plant communities.
