# Supplementary material for: The Patterns of Altitudinal Gradient Differentiation in the Morphological Traits of Calliptamus italicus (L.) (Orthoptera: Acridoidea) and Their Environmental Driving Mechanisms in the Desert Steppe in the Ili River Basin
Source: Insects. 2026 Apr 22;17(5):445. doi: 10.3390/insects17050445 (PMC13207455; doi:10.3390/insects17050445)
Supplement: Supplementary file 1 [file insects-17-00445-s001.zip › insects-4240956-supplementary.pdf]

Table S1: Measured morphological characters of Calliptamus italicus

Morphological character	Abbreviation	Definition and measurement method
Forewing area	FWA	Area of the right forewing, calculated by image analysis using Photoshop
Forewing length	FWL	Maximum length of the right forewing, measured with digital caliper (0.01 mm)
Forewing width	FWW	Maximum width of the right forewing, measured with digital caliper (0.01 mm)
Hindwing area	HWA	Area of the right hindwing, calculated by image analysis using Photoshop
Hindwing length	HWL	Maximum length of the right hindwing, measured with digital caliper (0.01 mm)
Hindwing width	HWW	Maximum width of the right hindwing, measured with digital caliper (0.01 mm)
Body length	BL	Distance from the front of the head to the tip of the abdomen
Head width	HW	Maximum width of the head including compound eyes
Head height	HH	Vertical height of the head
Pronotum length	PL	Maximum length of the pronotum
Pronotum width	PW	Maximum width of the pronotum
Pronotum height	PH	Vertical height of the pronotum

Morphological character	Abbreviation	Definition and measurement method
Hind tibia length	HTL	Length of the right hind tibia
Hind tibia width	HTW	Maximum width of the right hind tibia

Table S2: Morphological-adaptive significance of the 15 measured traits in relation to environment

No.	Trait	English name	Adaptive function	Environmental implication
1	Body length	Body length	Body size, heat dissipation efficiency, feeding capacity	Smaller body size at low temperature to reduce heat loss; larger body size at high temperature for better heat dissipation
2	Head width	Head width	Feeding efficiency, mouthpart development, sensory ability	Stable in arid environments; affected by vegetation structure
3	Pronotum length	Pronotum length	Thorax support, movement protection, flight assistance	Shorter at higher altitude/lower temperature to save energy
4	Forewing length	Forewing length	Flight capacity, dispersal distance, thermoregulation	Longer at high altitude to enhance flight and dispersal
5	Forewing width	Forewing width	Flight lift, gliding efficiency, body temperature regulation	Wider at high altitude to improve gliding and reduce energy cost
6	Forewing	Forewing	Flight efficiency, heat	Larger at high altitude for better

No.	Trait	English name	Adaptive function	Environmental implication
	area	area	dissipation area, defense protection	flight and heat dissipation
7	Hind femur length	Hind femur length	Jumping ability, predator escape, dispersal	Shorter at low temperature to lower energy consumption
8	Hind femur width	Hind femur width	Jumping strength, muscle attachment, stress resistance	Wider in arid environments to improve jumping stability
9	Hind tibia length	Hind tibia length	Body support, climbing ability, environmental perception	Shorter at high altitude to adapt to low temperature and hypoxia
10	Hind femur circumference	Hind femur circumference	Muscle development, movement endurance, cold tolerance	Smaller at low temperature to reduce metabolic cost
11	Forewing / body length ratio	Forewing/body length ratio	Flight efficiency, energy allocation, dispersal strategy	Higher at high altitude to prioritize flight capacity
12	Hind leg / body length ratio	Hind leg/body length ratio	Locomotion strategy, habitat adaptation, escape efficiency	Stable in arid environments; lower at low temperature
13	Head width / body length ratio	Head width/body length ratio	Feeding efficiency, energy allocation, environmental adaptation	Higher in poor vegetation areas to ensure feeding
14	Pronotum / body	Pronotum/body length ratio	Body structure, movement protection,	Lower at high altitude to reduce developmental energy consumption

No.	Trait	English name	Adaptive function	Environmental implication
	length ratio		developmental cost	
15	Femur / tibia length ratio	Femur/tibia length ratio	Jumping efficiency, movement balance, habitat adaptation	Stable in desert steppe to adapt to open habitats

Table S3: Species composition and abundance of grasshoppers along the elevation gradient

Family	Genus	Species	Altitude gradient 1 (700-1000)（m）	Altitude gradient 2 (1000-1300)	Altitude gradient 3 (>1300)	Total individuals
Acrididae	*Calliptamus*	*Calliptamus italicus*	936	1363	804	3103
Acrididae	*Calliptamus*	*Calliptamus barbarus*	220	243	89	552
Oedipodidae	*Oedipoda*	*Oedipoda miniata*	379	125	100	604
Oedipodidae	*Oedipoda*	*Oedipoda caerulescens*	9	27	72	108
Arcypteridae	*Dociostaurus*	*Dociostaurus tartarus*	423	173	183	779
Arcypteridae	*Dociostaurus*	*Dociostaurus kraussi*	22	167	409	598
Oedipodidae	*Oedaleus*	*Oedaleus decorus*	298	335	322	955
Arcypteridae	*Notostaur*	*Notostaurus*	19	55	2	76

	us	*albicornis*				
Oedipodidae	*Sphingonotus*	*Sphingonotus coerulipes*	25	-	-	25
Oedipodidae	*Sphingonotus*	*Sphingonotus maculatus*	6	-	-	6
Oedipodidae	*Sphingonotus*	*Sphingonotus petraeus*	-	2	-	2
Pamphagidae	*Egnatius*	*Egnatius longicornis*	8	20	-	28
Arcypteridae	*Omocestus*	*Omocestus haemorrhoidalis*	4	-	148	152
Arcypteridae	*Pararcyptera*	*Pararcyptera microptera*	2	-	10	12
Arcypteridae	*Chorthippus*	*Chorthippus albomarginatus*	1	2	83	86
Arcypteridae	*Chorthippus*	*Chorthippus dichrous*	-	1	1	2
Arcypteridae	*Chorthippus*	*Chorthippus biguttulus*	-	-	77	77
Acrididae	*Calliptamus*	*Calliptamus coelesyriensis*	-	22	6	28
Arcypteridae	*Euchorthippus*	*Euchorthippus pulvinatus*	-	-	3	3
Arcypteridae	*Stauroderus*	*Stauroderus scalaris*	-	1	7	8
Arcypteridae	*Asiotmethis*	*Asiotmethis zacharjini*	-	1	-	1
Oedipodidae	*Ramburiella*	*Ruriella amburiella turcomana*	-	6	6	12
Total	-	-	2352	2543	2322	7217

Table S4: Species composition of plant communities

Family	Genus	Species
Asteraceae	*Seriphidium*	*Seriphidium transiliense*
Fabaceae	*Sophora*	*Sophora alopecuroides*
Chenopodiaceae	*Ceratocarpus*	*Ceratocarpus arenarius*
Asteraceae	*Filago*	*Filago arvensis*
Chenopodiaceae	*Salsola*	*Salsola ruthenica*
Chenopodiaceae	*Kochia*	*Kochia prostrata*
Poaceae	*Aegilops*	*Aegilops tauschii*
Amaranthaceae	*Amaranthus*	*Amaranthus viridis*
Chenopodiaceae	*Chenopodium*	*Chenopodium album*
Chenopodiaceae	*Ceratoides*	*Ceratoides latens*
Polygonaceae	*Polygonum*	*Polygonum aviculare*
Convolvulaceae	*Convolvulus*	*Convolvulus arvensis*
Rosaceae	*Alchemilla*	*Alchemilla vulgaris*
Cyperaceae	*Carex*	*Carex spp.*
Primulaceae	*Androsace*	*Androsace umbellata*
Boraginaceae	*Lappula*	*Lappula myosotis*
Brassicaceae	*Malcolmia*	*Malcolmia africana*

Asteraceae	*Taraxacum*	*Taraxacum mongolicum*
Poaceae	*Phleum*	*Phleum pratense*
Poaceae	*Elymus*	*Elymus nutans*
Fabaceae	*Trigonella*	*Trigonella arcuata*
Asteraceae	*Chondrilla*	*Chondrilla pipiocoma*
Euphorbiaceae	*Euphorbia*	*Euphorbia pekinensis*
Asteraceae	*Artemisia*	*Artemisia gmelinii*
Rubiaceae	*Galium*	*Galium aparine*
Lamiaceae	*Phlomis*	*Phlomis umbrosa*
Poaceae	*Cynodon*	*Cynodon dactylon*
Saxifragaceae	*Saxifraga*	*Saxifraga stolonifera*
Asteraceae	*Cirsium*	*Cirsium japonicum*
Poaceae	*Festuca*	*Festuca ovina*
Fabaceae	*Medicago*	*Medicago sativa*
Poaceae	*Achnatherum*	*Achnatherum splendens*
Fabaceae	*Caragana*	*Caragana sinica*
Asteraceae	*Heteropappus*	*Heteropappus hispidus*
Asteraceae	*Artemisia*	*Artemisia desertorum*
Plantaginaceae	*Plantago*	*Plantago asiatica*
Asteraceae	*Achillea*	*Achillea millefolium*

Rubiaceae	*Rubia*	*Rubibiaceae*
Poaceae	*Avena*	*Avena fatua*
Poaceae	*Poa*	*Poa annua*
Zygophyllaceae	*Tribulus*	*Tribulus terrestris*
Chenopodiaceae	*Bassia*	*Bassia dasyphylla*
Poaceae	*Eragrostis*	*Eragrostis pilosa*
